# Timing‐dependent protective and therapeutic effects of ethyl pyruvate in trinitrobenzene sulfonic acid‐induced experimental colitis

**DOI:** 10.1113/EP093786

**Published:** 2026-06-17

**Authors:** Hatice Yorulmaz, Elif Yorulmaz, Elif Özkök, Suat Hayri Küçük, Erdem Sünger, Ayyub Ebrahimi, Beyza Yılmaz, Serdar Altınay

**Affiliations:** ^1^ Faculty of Medicine Haliç University, Eyup Istanbul Turkey; ^2^ Department of Gastroenterology University of Health Sciences Bağcılar Training and Research Hospital Istanbul Turkey; ^3^ Department of Neuroscience Aziz Sancar Institute of Experimental Medicine Istanbul University Istanbul Turkey; ^4^ Departments of Biochemistry Bağcılar Training and Research Hospital Istanbul Turkey; ^5^ Medical Oncology Clinic University of Istanbul Medipol Istanbul Turkey; ^6^ Molecular Biology and Genetics Haliç University Istanbul Turkey; ^7^ MRC Laboratory of Medical Sciences London UK; ^8^ Institute of Clinical Sciences Imperial College Faculty of Medicine London UK; ^9^ Institute of Biotechnology Gebze Technical University Kocaeli Turkey; ^10^ Department of Pathology Faculty of Medicine University of Health Sciences Istanbul Turkey

**Keywords:** colitis, ethyl pyruvate, rat, trinitrobenzene sulfonic acid

## Abstract

Ulcerative colitis (UC) involves excessive inflammation, extracellular matrix remodelling and oxidative stress. Ethyl pyruvate (EP) exhibits anti‐inflammatory effects, but the comparative efficacy of prophylactic versus therapeutic administration and timing‐dependent molecular responses remains unclear. The objective of this work was to evaluate timing‐dependent protective and therapeutic effects of EP in trinitrobenzene sulfonic acid (TNBS)‐induced experimental colitis, focusing on inflammatory mediators, matrix metalloproteinases (MMPs), oxidative status and gene expression. Rats were randomly assigned to Control, EP, Colitis, Pre‐colitis (EP before TNBS) and Post‐colitis (EP after TNBS) groups. Colonic injury was assessed macroscopically and histopathologically. Tissue levels of matrix metalloproteinase (MMP)‐2, MMP‐9, interleukin (IL)‐1, IL‐17, IL‐10, nuclear factor‐κB (NF‐κB), inducible nitric oxide synthase (iNOS) and total antioxidant capacity (TAC) were measured using ELISA kits. Gene expression of MMP‐9, cytokines, NF‐κB, high mobility group box 1 (HMGB1), iNOS and interferon‐γ was analysed by quantitative real‐time PCR. TNBS induced severe colonic injury with upregulation of pro‐inflammatory cytokines, NF‐κB signalling, MMP‐2/9 and oxidative imbalance (*P *< 0.01). EP attenuated tissue damage in both prophylactic and therapeutic settings. Post‐colitis (therapeutic) EP administration produced greater suppression of MMP‐2, MMP‐9, NF‐κB, HMGB1 and iNOS, along with superior restoration of TAC (*P *< 0.05), indicating timing‐dependent modulation of inflammatory and matrix degradation pathways. EP exerts both preventive and therapeutic effects in TNBS‐induced colitis by suppressing inflammatory signalling, regulating extracellular matrix remodelling and enhancing antioxidant defences. Therapeutic administration is more effective than prophylactic treatment, highlighting the importance of timing in optimizing EP's efficacy in inflammatory bowel disease.

## Abstract (highlights)

- **What is the central question of this study?**
  Does ethyl pyruvate (EP) exert timing‐dependent protective and therapeutic effects in trinitrobenzene sulfonic acid‐induced experimental colitis and does its efficacy differ between prophylactic and therapeutic administration?
- **What is the main finding and its importance?**
  EP reduces inflammation, oxidative stress and extracellular matrix degradation in experimental colitis. Therapeutic (post‐colitis) administration shows greater efficacy than prophylactic treatment by more strongly suppressing nuclear factor‐κB signalling, matrix metalloproteinase‐2/9 expression and inflammatory mediators while improving antioxidant capacity. These findings highlight the importance of treatment timing in optimizing EP efficacy for inflammatory bowel disease.

## 1 INTRODUCTION

Ulcerative colitis (UC) is an inflammatory bowel disease (IBD) characterized by symptoms such as mucosal inflammation, rectal bleeding and diarrhoea (Du & Ha, 2020). In UC, lymphocytes and macrophages come to the infection site, resulting in dysregulation of the immune response, the elevation of high mobility group box 1 protein (HMGB1), imbalanced production of nuclear factor κB (NF‐κB) and proinflammatory cytokines such as interleukin (IL)‐12, IL‐1, IL‐23 and tumour necrosis factor‐α (TNF‐α). High levels of inducible nitric oxide synthase (iNOS) and cyclooxygenase‐2 (COX‐2) may damage the colon mucosa by disrupting the balance between the oxidant/antioxidant system (Zhu et al., 2019). Also, reactive oxygen species (ROS) in inflamed mucosa may contribute to tissue damage by increasing cytokine production (Jena et al., 2012). Metalloproteinases (MMPs) may play a role in the IBD pathogenesis by causing an inflammatory response, degradation of the extracellular matrix and excessive damage to intestinal tissues (Koelink et al., 2014). Changes in MMP expression patterns have been reported in IBD, suggesting a role for these molecules in intestinal inflammation and tissue remodeling (Mäkitalo et al., 2012) Supporting information.

Ethyl pyruvate (EP) is a simple derivative of pyruvate, the end product of glycolysis and a precursor for the Krebs cycle. EP is an effective scavenger of hydrogen peroxide and other ROS and an effective anti‐inflammatory agent (Yang et al., 2016). EP prevents organ damage in experimental models of severe sepsis, acute pancreatitis and traumatic brain injury by exerting anti‐inflammatory impacts mediated through the blocking of NF‐κβ and the release of TNF‐α, IL‐1β and IL‐6 (Koprivica et al., 2022). Few studies have been conducted on the effects of EP on intestinal tissue in UC. In experimental colitis models, EP has been administered at different doses and timings, mostly in early‐phase dextran sodium sulfate (DSS) colitis. Comparative studies on timing‐dependent effects in trinitrobenzene sulfonic acid (TNBS)‐induced colitis are lacking.

The studies showed the effects of EP in different doses in the early phase of UC. In experimental colitis models, EP has been administered at different doses and timings, mostly focusing on early‐phase DSS colitis, while comparative timing‐dependent studies in TNBS‐induced colitis are lacking (Algieri et al., 2016; Guo et al., 2015). Although the effects of EP in many inflammatory conditions were examined in the literature reviews, we could not find any study on the effects of pre‐colitis application. The TNBS colitis model has a short experimental period and much similarity to human IBD. It is a suitable model for investigating the pathophysiology and new treatment methods of colonic IBD due to the long‐term persistence of inflammation and ulceration (Yildiz et al., 2015). Therefore, our study investigates prophylactic versus therapeutic EP administration in TNBS‐induced colitis, highlighting timing‐dependent molecular modulation of inflammatory mediators, MMPs, NF‐κB, HMGB1, iNOS and antioxidant capacity.

## 2 METHODS

### 2.1 Ethical approval and experimental design

Ethical approval for this study was obtained from the Bağcılar Training and Research Hospital Animal Experiments Local Ethics Committee (Approval No: 2021/95; Assoc. Prof. Duygu Sultan Oran). All experimental procedures complied with institutional and international guidelines for animal care and use and with the policies of *Experimental Physiology*. Adult male Wistar Hannover rats weighing 180–230 g were used to minimize hormonal variability in the study. The rats were housed in special cages under a 12 h light–dark cycle in a ventilated room with constant temperature conditions. Animals had free access to standard laboratory chow and water ad libitum throughout the experimental period. A priori power analysis was performed using G*Power software (version 3.1) to determine the minimum sample size required to detect differences between groups. The analysis was based on one‐way ANOVA (fixed effects, omnibus test) with an α‐level of 0.05 and statistical power of 80% (β = 0.20). Effect size estimation was based on previously published studies using TNBS‐induced colitis models reporting comparable outcome measures. Based on this analysis, a sample size of seven rats per group was considered sufficient to detect biologically and statistically meaningful differences in primary molecular endpoints. Rats were randomly assigned to five groups using a simple randomization method. Each rat was allocated to a group by drawing lots to ensure equal probability of assignment: Control (*n* = 7), EP (*n* = 7), Colitis (*n* = 7), Pre‐colitis (*n* = 7) and Post‐colitis (*n* = 7).

Control group: no colitis was induced in this group; normal saline was administered rectally.

EP group: EP (E47808, Sigma‐Aldrich, St Louis, MO, USA; 60 mg/kg) was administered intraperitoneally (i.p.) once daily for two consecutive days. The dose (60 mg/kg, i.p.) was selected based on preliminary experiments and its known pharmacological safety profile in animals, aiming to achieve sufficient systemic levels for anti‐inflammatory and antioxidant effects.

Colitis group: after 12 h of fasting, colitis was induced under anaesthesia. Anaesthesia was induced by ketamine (35 mg/kg, i.p.) and xylazine (5 mg/kg, i.p.), and adequate depth of anaesthesia was confirmed by absence of pedal withdrawal reflex. TNBS (50 mg; Sigma‐Aldrich, P2297) dissolved in 50% ethanol (1.5 mL) was then administered intrarectally using a size 6 soft feeding tube.

Pre‐colitis group: EP (60 mg/kg, i.p.) was administered once daily for two days prior to TNBS administration. TNBS was applied one hour after the first EP injection.

Post‐colitis group: colitis was first induced with TNBS as described above. EP (60 mg/kg, i.p.) treatment was initiated 1 h after TNBS administration and continued once daily for two consecutive days.

All animals were euthanized 48 h after TNBS administration under deep anaesthesia induced by ketamine (90 mg/kg, i.p.) and xylazine (10 mg/kg, i.p.). Death was ensured by exsanguination via cardiac puncture. Blood samples (5 mL) were collected via cardiac puncture prior to tissue harvesting. The large intestine was then excised for further analyses.

### 2.2 Biochemical procedures

The concentrations of alanine aminotransferase (ALT) and aspartate aminotransferase (AST) were measured using an autoanalyzer (Cobas, Roche Diagnostics, Basel, Switzerland). Blood leukocytes and neutrophils were analyzed using a hematology analyzer (LH 780, Beckman Coulter, Brea, CA, USA).

### 2.3 Enzyme‐linked immunosorbent assay (ELISA) assessment

Rat intestinal MMP‐9, MMP‐2, IL‐17, IL‐1, IL‐10, NF‐κB, TAC, and iNOS levels were measured using ELISA kits (BT‐Lab, Shanghai, China; Cat Nos: E0321Ra, E0315Ra, 115Ra, E0107Ra, E0108Ra, E0287Ra, E1710Ra, E0740Ra) according to the manufacturer's instructions.

### 2.4 Histopathological analysis

After staining the abdomen with povidone–iodine, laparotomy was performed with a xiphopubic midline incision, and exploration was performed. The colon segment was resected from the distal rectum as low as possible, and approximately 8 cm of the colon segment was removed. The removed colon resection material was opened longitudinally, and the faecal content was gently cleaned with 0.9% saline. Macroscopic grading of colonic mucosa in fresh tissue was scored from non‐colonic damage to major ulcer and perforation at six levels as previously described by Millar et al. (1996); 0, no colonic damage; 1, hyperaemia but no ulcer; 2, linear ulcer but no colonic wall thickening; 3, linear ulcer and colonic wall thickening at one area; 4, colonic ulcer at multiple areas; 5, major ulcer and perforation. The tissues obtained from rats were fixed in a 10% buffered formaldehyde solution for 24 h. Fixed samples were embedded in paraffin blocks after routine tissue follow‐up. Sections of 4 µm thickness were cut and taken using a microtome. Finally, the sections were stained with haematoxylin and eosin (H&E), and histopathological changes were detected by light microscopy (Nikon Ni 50, Tokyo, Japan). Changes in the colonic mucosa were scored as previously described by Yamamoto et al. (2000). Sections were analysed considering the severity and extent of oedema, apoptosis, necrosis, vascular congestion, inflammatory infiltration, thrombosis and partial detachment in epithelial cells. Scoring was done between 0 and 3 degrees (Yamamoto et al., 2000). Histopathological evaluation was performed by a single histopathologist who was blinded to the experimental groups to minimize observer bias.

### 2.5 RNA isolation and cDNA synthesis

Fragments of approximately 50 mg were obtained from the large intestine tissues contained in RNAlater (Thermo Fisher Scientific, Waltham, MA, USA) using sterile surgical equipment. Then, these tissue pieces were cut into smaller sizes and homogenized for 30 seconds (frequency 1/s) using a Retsch MM 400 device (Retsch, Haan, Germany) with 0.5 cm metal beads. After homogenization, total RNA was extracted using a Quick‐RNA Miniprep Kit (Zymo Research, Irvine, CA, USA, R1055) according to the manufacturer's instructions. The concentration of RNAs was calculated with a microplate reader (Thermo Fisher Scientific, MultiskanGo). cDNA was synthesized from 1 µg RNA in a thermal cycler (Bio‐Rad, T100, Hercules, CA, USA) using a cDNA synthesis kit (Bioline, London, UK; Cat No: BIO‐65054) according to the manufacturer's instructions.

### 2.6 Quantitative real time‐PCR

Quantitative gene expression analysis was performed using the Real Time‐PCR (RT‐PCR) Detection System (Bio‐Rad, CFX96™ Connect) with the SensiFAST™ SYBR® No‐ROX Kit (Bioline, BIO‐98020). The mRNA levels of *MPP‐9*, *IL‐17*, *IL‐1*, *IL‐12*, *HMGB1*, *iNOS*, *NF‐κB*, *TNF‐α* and interferon‐γ (*IFN‐γ*) genes were calculated using the $2^{-\Delta \Delta C_{t}}$ algorithm, and the results were normalized to the *β‐Actin* housekeeping gene. The oligonucleotide primers used in this study (Table  1) were designed using Primer3 and NCBI databases and synthesized at Sentromer DNA Technology (Ankara, Turkey).

**TABLE 1:** Primers and sequences for selected inflammation‐related genes.

Gene of interest	Forward primers (5′–3′)	Reverse primers (5′–3′)
*MMP‐9*	AGGATGGTCTACTGGCACAC	GTGCAGGACAAATAGGAGCG
*IL‐1*	GGGATGATGACGACCTGCTA	TGTCGTTGCTTGTCTCTCCT
*IL‐17*	CAGCGGTACTCATCCCTCAA	TATCAGGGTCCTCATTGCGG
*IL‐12*	CAGAGGGGACAACAAGGAGT	TCCACCTGCCGAGAATTCTT
*iNOS*	GTTTGACCAGAGGACCCAGA	GTGAGCTGGTAGGTTCCTGT
*NF‐κB*	AGAGGATGTGGGGTTTCAGG	GCTGAGCATGAAGGTGGATG
*HMGB1*	TCGGCCTTCTTCCTCTTCTG	TCAGCCTTGACAACTCCCTT
*IFN‐γ*	TGCAGAGCCAAATTGTCTCC	TGCTTTGCGTTGGACATTCA
*TNF‐α*	GTCAACCTCCTCTCTGCCAT	CCAAAGTAGACCTGCCCAGA

### 2.7 Statistical analysis

All statistical analysis of the study was performed with IBM SPSS Statistics (Version 22.0; IBM Corp., Armonk, NY, USA). Data are presented as means ± standard deviation (SD). Normality distributions of the data were evaluated with the Shapiro–Wilk test. All parametric data were evaluated with a one‐way analysis of variance (ANOVA) followed by Tukey's *post hoc* test, and non‐parametric data were evaluated with Kruskal–Wallis analysis of variance and the Mann–Whitney *U*‐test. A value of *P *< 0.05 was considered statistically significant.

## 3 RESULTS

### 3.1 Biochemical findings

Leukocyte and neutrophil counts were significantly higher in the Colitis group compared with Control, EP and Post‐colitis groups (leukocytes *P *< 0.0001; neutrophils *P *< 0.0001), with leukocytes also higher in the Colitis than in the Pre‐colitis group (*P* = 0.002). Both parameters were lower in the Pre‐ and Post‐colitis groups compared with the Colitis group. ALT levels were elevated in the Colitis versus Control and EP groups (*P *< 0.0001), with significant differences between Colitis and Pre‐colitis (*P* = 0.044) and Colitis and Post‐colitis groups (*P* = 0.007). AST levels were higher in the Colitis group compared with the Control, EP and Post‐colitis groups (*P *< 0.0001) and were lower in the Pre‐ and Post‐colitis groups. Data are presented as means ± SD (*n* = 7 animals per group) (Table 2).

**TABLE 2:** Comparison of leukocyte, neutrophil, ALT, and AST levels across experimental groups.

Groups	Leukocytes (×1000/µL)	Neutrophils (×1000/µL)	ALT (U/L)	AST (U/L)
Control	7.44 ± 0.92	2.18 ± 0.27	53.68 ± 4.71	96.94 ± 13.43
EP	7.32 ± 0.97	2.84 ± 0.40	52.21 ± 7.02	94.27 ± 7.06
Colitis	10.4 ± 1.30	5.92 ± 0.87	67.38 ± 4.43	145.9 ± 10.32
Pre‐colitis	8.32 ± 0.50	2.95 ± 0.46	59.45 ± 4.45	106.4 ± 7.31
Post‐colitis	8.02 ± 0.84	2.51 ± 0.44	57.51 ± 3.82	104.51 ± 7.74

Leukocyte, neutrophil, ALT and AST were higher in colitis versus control, EP and post‐colitis (leukocytes *P *< 0.0001; neutrophils *P *< 0.0001; ALT *P *< 0.0001; AST *P *< 0.0001), with colitis vs. pre‐colitis (leukocytes *P* = 0.002; ALT *P* = 0.044) and colitis vs. post‐colitis (ALT *P* = 0.007); all were lower in pre‐ and post‐colitis.

### 3.2 ELISA findings

Tissue MMP‐9 levels were significantly increased in the Colitis and Pre‐colitis groups compared with the Control, EP and Post‐colitis groups (*P *< 0.0001 for all comparisons). MMP‐9 values were lower in the Post‐colitis group than in the Pre‐colitis and Colitis groups (*P *< 0.0001). MMP‐2 levels were higher in the Colitis and Pre‐colitis groups than in the Control, EP and Post‐colitis groups (Control and EP: *P *< 0.0001; vs. Post‐colitis: *P* = 0.002 and *P* = 0.004, respectively). MMP‐2 levels were also lower in the Post‐colitis group than in the Colitis and Pre‐colitis groups (*P* = 0.002 and *P* = 0.004, respectively).

Tissue NF‐κB levels were significantly increased in the Colitis group compared with the Control and EP groups (*P *< 0.0001 for both comparisons). A similar increase was also observed in the Pre‐colitis group, which showed higher NF‐κB levels compared with the Control (*P *< 0.0001) and EP (*P* = 0.001) groups. In contrast, NF‐κB levels were significantly reduced in the Post‐colitis group compared with the Colitis group (*P* = 0.015). iNOS levels were significantly increased in the Colitis, Pre‐colitis and Post‐colitis groups compared with the Control and EP groups (*P *< 0.001). Levels of TAC increased in the Post‐colitis group compared with the Control (*P *< 0.005). IL‐17 levels were significantly increased in the Colitis group compared with the Control, EP, Pre‐colitis and Post‐colitis groups (Colitis vs. Control: *P* = 0.004; Colitis vs. EP and Colitis vs. Pre‐colitis: *P *< 0.0001; Colitis vs. Post‐colitis: *P* = 0.001).

**FIGURE 1:**
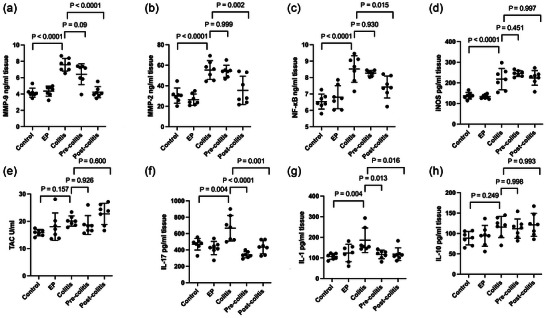
ELISA analysis of tissue MMP‐9, MMP‐2, NF‐κB, iNOS, TAC, IL‐17, IL‐1 and IL‐10 levels. (a) MMP‐9 levels. Levels were higher in the Colitis and Pre‐colitis groups than in the Control, EP and Post‐colitis groups (all *P *< 0.0001) and lower in the Post‐colitis group than in the Pre‐colitis and Colitis groups (all *P *< 0.0001). (b) MMP‐2 levels differed among groups, with higher values in the Colitis and Pre‐colitis groups and lower values in the Post‐colitis group (Control, EP and Post‐colitis comparisons: *P *< 0.0001; additional comparisons: *P* = 0.0002 and *P* = 0.004). (c) NF‐κB levels. NF‐κB levels were increased in the Colitis and Pre‐colitis groups compared with the Control and EP groups (Colitis: *P *< 0.0001; Pre‐colitis: Control *P *< 0.0001, EP *P* = 0.001) and reduced in the Post‐colitis group compared with the Colitis group (*P* = 0.015). (d) iNOS levels. iNOS levels were increased in the Colitis, Pre‐colitis and Post‐colitis groups compared with the Control and EP groups (*P *< 0.0001 for all comparisons). (e) TAC levels. TAC levels were significantly higher in the Post‐colitis group compared with Control (*P* = 0.005). (f) IL‐17 levels. IL‐17 levels were increased in the Colitis group compared with the other groups (Control: *P* = 0.004; EP and Pre‐colitis: *P *< 0.0001; Post‐colitis: *P* = 0.001). (g) IL‐1 levels. IL‐1 levels were increased in the Colitis group compared with all groups (Control: *P* = 0.004; EP: *P* = 0.031; Pre‐colitis: *P* = 0.013; Post‐colitis: *P* = 0.016). (h) IL‐10 levels. IL‐10 levels did not show statistically significant differences among the groups (all comparisons *P* > 0.05). Data are presented as means ± SD (*n* = 7 animals per group). Due to figure clarity limitations, only the statistically significant comparisons between the Colitis group and the Control, Pre‐colitis and Post‐colitis groups are indicated within the figure.

IL‐1 levels were significantly increased in the Colitis group compared with the Control, EP, Pre‐colitis and Post‐colitis groups (Control: *P* = 0.004; EP: *P* = 0.031; Pre‐colitis: *P* = 0.013; Post‐colitis: *P* = 0.016). IL‐10 levels did not differ significantly between the Colitis and Pre‐colitis groups (*P* = 0.0993) or between the Colitis and Post‐colitis groups (*P* = 0.0998). No other pairwise comparisons involving the Colitis group reached statistical significance (all *P* > 0.050; Figure 1).

### 3.3 Histopathological score findings

**FIGURE 2:**
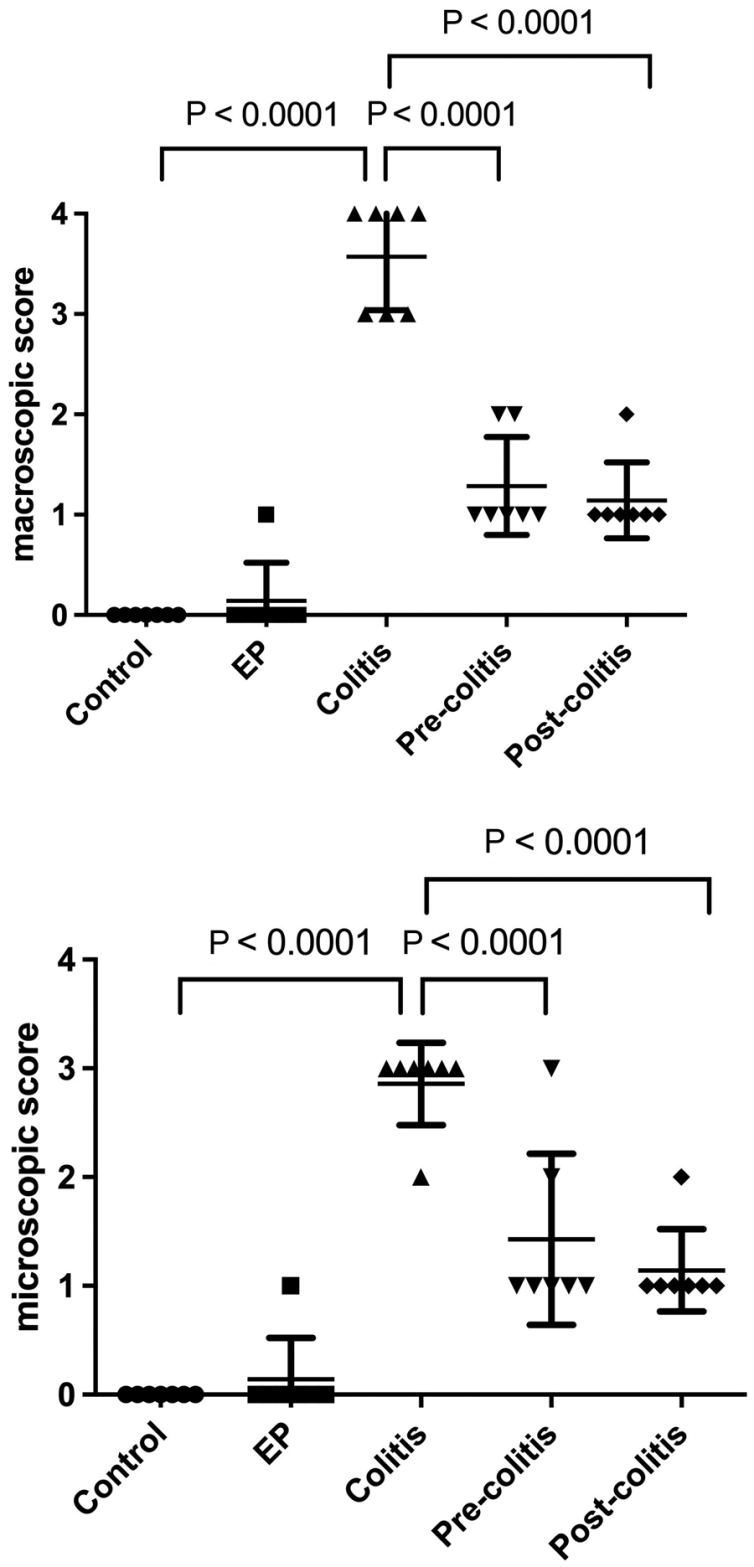
Distribution of macroscopic and microscopic colonic damage scores across experimental groups. Data are presented as means ± SD with individual data points shown (*n* = 7 animals per group). According to macroscopic and microscopic scores, the Colitis group showed significantly higher scores compared with all groups (*P *< 0.0001). Both Pre‐colitis and Post‐colitis groups showed significantly lower scores compared with the Colitis group (*P *< 0.0001).

**FIGURE 3:**
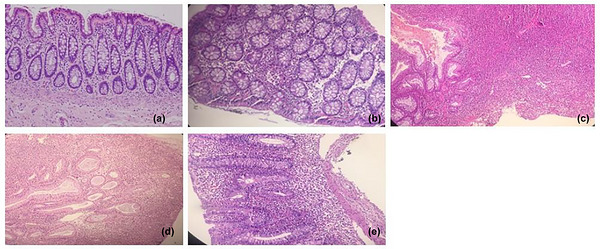
Microscopic presentation of experimental groups. (a) Section of large intestine tissue from the control group stained with H&E, ×100 magnification. (b) Section of large intestine tissue from the EP group (H&E; ×200). (c) Section of large intestine tissue from Colitis group shows surface epithelial damage and significant ulceration in the colitis group (H&E; ×100). (d) Section of large intestine tissue from the Pre‐colitis group shows marked inflammation of the lamina propria and dilatation of the crypts in the Pre‐colitis group (H&E; ×100). (e) Clusters of polymorphonuclear leukocytes concentrated in places in the crypt epithelium and significant inflammation in the lamina propria are seen in the Post‐colitis group (H&E; ×100).

There were no macroscopic or microscopic changes in the control rats (macroscopic and microscopic score range: 0–0). One of the rats in the EP group showed hyperaemia macroscopically, and minimal oedema and vascular congestion on microscopy (macroscopic and microscopic score range: 0–1 (0.33 ± 0.57)). Four of the rats in the Colitis group had macroscopically multiple fields of ulcers, while three had a linear ulcer and wall thickening (macroscopic score range: 3–4 (3.57 ± 0.53)). In the Colitis group, with microscopically varying severities, there were signs of colitis accompanied by oedema, necrosis, inflammatory infiltration, apoptosis, vascular conjunction, thrombosis and decomposition in apical epithelial cells. The colitis diagram showed the distance up to the anal mucosa. Six of the rats scored 3, while only one scored 1 (microscopic score range: 2–3 (2.85 ± 0.37)). In the Pre‐colitis group, only two of the rats had a macroscopically linear ulcer and wall thickening, while the other five had mild hyperaemia not accompanied by ulcer (macroscopic score range: 1–2 (1.28 ± 0.48)). In the Pre‐colitis group, only one rat had a microscopic score of 3 and colitis, while the other five rats had a score of 1, and one rat scored 2 with colitis (microscopic score range: 1–3 (1.42 ± 0.78)). Only one of the Post‐colitis rats had macroscopically linear ulcers and wall thickening, while the other six had mild hyperaemia not accompanied by ulcers. In the Post‐colitis group, only one rat had a score of 2 colitis observations under the microscope, while the other 6 rats had a score of 1 colitis (macroscopic and microscopic score range: 1–2 (1.14 ± 0.37)). In the Colitis, Pre‐colitis and Post‐colitis groups, the macroscopic and microscopic scores were higher than in other groups (*P *< 0.001). However, the score values were significantly lower in the Pre‐ and Post‐colitis groups compared to the Colitis group (*P *< 0.001). No statistically significant differences were observed between the macroscopic and microscopic scores of the Pre‐ and Post‐colitis groups (*P* > 0.050, Figures 2 and 3).

### 3.4 Gene expression results

**FIGURE 4:**
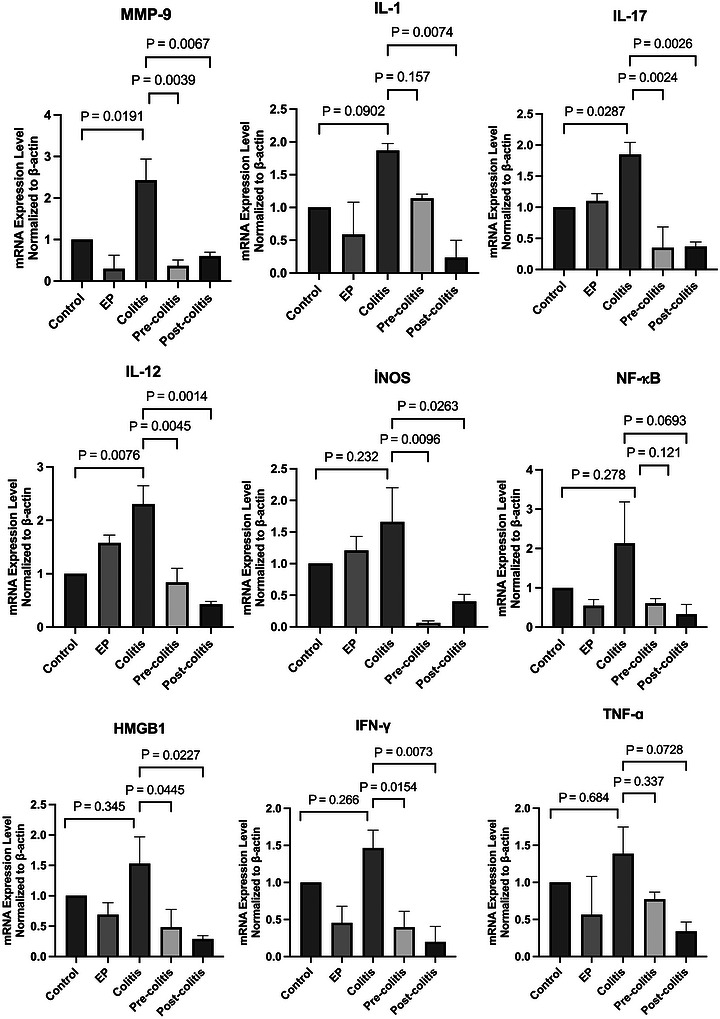
Analysis of the gene expression profile of inflammatory‐related genes. Analysis of the gene expression profile showed that the expression of *MMP‐9*, *IL‐1*, *IL‐17*, *IL‐12*, *iNOS*, *NF‐κB*, *HMGB1*, *IFN‐γ* and *TNF‐α* genes increased in the Colitis group compared to the Control group (*P* = 0.191 for *MMP‐9*, *P* = 0.0902 for *IL1*, *P* = 0.0287 for *IL‐17*, *P* = 0.0076 for *IL‐12*, *P* = 0.232 for *iNOS*, *P* = 0.278 for *NF‐κB*, *P* = 0.345 for *HMGB1*, *P* = 0.266 for *IFN‐γ* and *P* = 0.684 for *TNF‐α*) and the expression of these genes decreased in the Pre‐colitis group (*P* = 0.0039 for *MMP‐9*, *P* = 0.157 for *IL‐1*, *P* = 0.0024 for *IL‐17*, *P* = 0.0045 for *IL‐12*, *P* = 0.0096 for *iNOS*, *P* = 0.121 for *NF‐κB*, *P* = 0.0445 for *HMGB1* and *P* = 0.0154 for *IFN‐γ* and *P* = 0.337 for *TNF‐α*) and Post‐colitis group (*P* = 0.0067 for *MMP9*, *P* = 0.0074 for *IL‐1*, *P* = 0.0026 for *IL‐17*, *P* = 0.0024 for *IL‐12*, *P* = 0.0263 for iNOS, *P* = 0.121 for *NF‐κB*, *P* = 0.0693, *P* = 0.0227 for *HMGB1*, *P* = 0.0073 for *IFN‐γ* and *P* = 0.0728 for *TNF‐α*) compared to the Colitis group. Data represent the mean mRNA expression level of selected genes from two independent biological replicates, normalized to *β‐Actin*.

As a result of the qRT‐PCR, we performed an analysis to compare the expressions of various inflammatory genes. Our analysis demonstrated that, compared to the Control group, mRNA levels of *MMP‐9*, *IL‐1*, *NF‐κB*, *HMGB1*, *IFN‐γ* and *TNF‐α* genes were decreased, whereas *IL‐17*, *IL‐12* and *iNOS* expression levels were increased in the EP group. Our gene expression analysis results showed that the expression of all the genes we studied was increased in the Colitis group compared to the Control group and decreased in the Pre‐colitis and Post‐colitis groups compared to the Colitis group (Figure 4).

## 4 DISCUSSION

Our findings demonstrate that EP exerts timing‐dependent protective and therapeutic effects in TNBS‐induced colitis by modulating inflammatory signalling, matrix remodelling and oxidative pathways in the large intestine. Intrarectal administration of TNBS triggers cell‐mediated immune responses, causing histological changes resembling human UC, including leukocyte infiltration, oedema and ulceration (de Almeida et al., 2013). In our study, leukocyte, neutrophil and liver enzyme levels were elevated in the Colitis group, while both pre‐ and post‐colitis EP administration significantly reduced these markers. The biochemical results of our study demonstrate that experimental colitis induced significant inflammatory alterations, while EP treatment reduced these changes.

Excessive production of pro‐inflammatory cytokines and ROS contributes to intestinal damage in UC (Dulai & Jairath, 2018). Increased proinflammatory cytokines such as TNF‐α, IFN‐γ, IL‐6 and IL‐1β in UC may trigger apoptosis, which plays a role in damage in in vivo colitis models (Guan & Zhang, 2017). We observed increased NF‐κB expression in the colitis model. Both the Colitis and Pre‐colitis groups showed higher NF‐κB levels compared with the Control and EP groups, with no significant difference between them. In contrast, NF‐κB levels were reduced in the Post‐colitis group compared with the Colitis group, suggesting a modulatory effect of EP after disease induction. EP has been previously shown to inhibit NF‐κB activation and upregulate anti‐inflammatory cytokines such as IL‐10 (Davé et al., 2009; Miljković et al., 2015; Yu et al., 2010). EP has been shown to inhibit HMGB1 expression levels in both serum and colon tissue and ameliorate the disease by reducing Th1/Th17 activation in a mouse model of IBD (Guo et al., 2015). Our results align with these findings, showing that post‐colitis EP administration restored IL‐10 levels and suppressed pro‐inflammatory mediators.

MMP‐2 and MMP‐9 are key mediators of extracellular matrix degradation in colitis (Matusiewicz et al., 2014; Olsen et al., 2007). MMP‐9 and MMP‐2 have been shown to be consistently increased in different in vivo colitis models, such as T cell‐mediated colitis, TNBS‐induced colitis and human colitis (Marshall et al., 2015; Matusiewicz et al., 2014). It was shown that EP reduces MMP‐9 by inhibition of the mitogen‐activated protein kinase/extracellular signal‐regulated kinase pathway in the sepsis model (Lee & Kim, 2011). In our study, EP was associated with lower MMP‐9 levels in the EP‐treated group compared with the Colitis group, while no significant difference was observed between the Colitis and Pre‐colitis groups. In contrast, EP treatment after colitis induction resulted in decreased MMP‐2 and MMP‐9 levels in the Post‐colitis group, suggesting a more pronounced modulatory effect when administered after disease induction. Similarly, TAC levels increased most prominently in the Post‐colitis group, highlighting enhanced antioxidant defence. In addition, it has previously been shown that the intestinal mucosa's defence mechanism could be affected significantly by the overproduction of ROS and nitrogen metabolites (Liu et al., 2020). It was observed that the levels of iNOS in the tissue significantly increased in the Colitis, Pre‐colitis and Post‐colitis groups compared to the Control and EP groups.

EP scavenges ROS and has also been determined to decrease lipid peroxidation with the blocking of myeloperoxidase or malondialdehyde activity (Luan et al., 2013). In our study, it was found that there was an increase in TAC in all experimental groups compared to the Control group, and antioxidant activity increased the most in the Post‐colitis group. This increase in TAC suggests that EP enhances the overall antioxidant defence system, thereby contributing to the reduction of oxidative stress. The improved antioxidant status may also play a role in promoting mucosal healing and limiting tissue damage in colitis, particularly when administered therapeutically.

EP has previously been shown to be an effective compound in treating of intestinal infections and in repairing tissue or cell damage by reducing the secretion and expression of inflammatory factors (Zhao et al., 2018). Somani et al. showed that in acetic acid‐induced colitis, deteriorations such as epithelial architecture destruction, submucosal oedema, loss of crypt and goblet cells, and infiltration of neutrophils and lymphocytes into the mucosal and submucosal parts occur (Somani et al., 2014). Gures et al. proposed that EP might be a useful agent for the treatment and/or attenuation of the symptoms of IBDs (Gures et al., 2014). Our histopathological evaluations revealed that pre‐ and post‐EP administration decreased macroscopic and microscopic damage scores in TNBS‐induced colitis. NF‐κB causes high levels of expression of TNF‐α, which enables the production of IL‐1, IL‐12, IFN‐γ and MMPs (Varthya et al., 2021). High levels of NF‐κB lead to increased expression of iNOS and nitrative stress in colitis and cause high‐level expression of TNF‐α, which enables the production of IL‐1, IL‐12, IFN‐γ and MMPs (Gochman et al., 2012). NF‐κB, HMGB1 and iNOS are closely interconnected in the inflammatory process of colitis. HMGB1 can activate NF‐κB signalling, which in turn promotes iNOS expression, leading to increased nitric oxide production and oxidative stress. Therefore, the concurrent reduction of these markers by EP suggests suppression of a key inflammatory signalling axis. Histopathological evaluation confirmed that EP reduced colonic damage in both prophylactic and therapeutic settings, with post‐colitis administration showing lower macroscopic and microscopic scores. The inconsistency between the gene and protein levels in some genes such as iNOS and consequently the effect of EP on colitis treatment and/or protective effects may be because of the mRNA–protein correlations. ELISA analysis demonstrated significant increases in IL‐1 and IL‐17 protein levels in the colitis group, while no significant differences were observed in IL‐10 levels. In contrast, qRT‐PCR analysis showed no significant changes in the mRNA expression levels in the EP group compared to the control. These findings indicate that protein‐level alterations are not always accompanied by corresponding transcriptional changes, suggesting post‐transcriptional regulation or temporal differences in gene expression (Buccitelli & Selbach, 2020; Greenbaum et al., 2003). Overall, post‐colitis EP treatment exerted superior effects on both molecular and tissue outcomes, emphasizing its stronger therapeutic potential compared to pre‐colitis (prophylactic) application.

Compared to previous studies focusing on early‐phase DSS colitis, our work provides novel insight into timing‐dependent effects of EP in the TNBS model, highlighting the translational relevance of post‐injury administration in IBD therapy.

Our study demonstrates that EP exerts timing‐dependent protective and therapeutic effects in TNBS‐induced colitis. Post‐colitis (therapeutic) administration showed superior suppression of inflammatory mediators, matrix MMPs and oxidative stress, accompanied by improved histopathological outcomes. These findings highlight the importance of treatment timing and support EP as a potential timing‐sensitive adjunctive therapy for IBD.

## AUTHOR CONTRIBUTIONS

Elif Yorulmaz, Hatice Yorulmaz and Erdem Sünge: conceived the idea and design of the study. Suat Hayri Küçük, Elif Özkök, Ayyub Ebrahimi, Beyza Yılmaz, Serdar Altınay and Elif Yorulmaz: contributed to data collection. Elif Yorulmaz, Hatice Yorulmaz and Erdem Sünge. Data analysis was performed by Hatice Yorulmaz, Elif Özkök, Ayyub Ebrahimi, Beyza Yılmaz, Serdar Altınay. Writing–original draft. Elif Yorulmaz, Hatice Yorulmaz, Elif Özkök, Ayyub Ebrahimi, Beyza Yılmaz, Serdar Altınay. Writing–review and editing. All authors contributed to the development of the submitted manuscript. All authors have read and approved the final version of this manuscript and agree to be accountable for all aspects of the work in ensuring that questions related to the accuracy or integrity of any part of the work are appropriately investigated and resolved.

## CONFLICT OF INTEREST

None declared.

## Supporting information



Supplementary Tables S1. Raw Data Underlying Histopathological Scores, ELISA, and Biochemical Analyses.

Supplementary Tables S2. Raw Data Underlying Gene Expression Analyses.

## Data Availability

Data presented in this manuscript is available by contacting the corresponding author.
